# Protection against Staphylococcus aureus Colonization and Infection by B- and T-Cell-Mediated Mechanisms

**DOI:** 10.1128/mBio.01949-18

**Published:** 2018-10-16

**Authors:** Fan Zhang, Olivia Ledue, Maria Jun, Cibelly Goulart, Richard Malley, Ying-Jie Lu

**Affiliations:** a Department of Medicine, Division of Infectious Diseases, Boston Children’s Hospital, Harvard Medical School, Boston, Massachusetts, USA; b Centro de Biotecnologia, Instituto Butantan, São Paulo, Brazil; Emory University; Cedars-Sinai Medical Center; University of Greifswald

**Keywords:** B-cell responses, *Staphylococcus aureus*, T-cell immunity, adaptive immunity, vaccines

## Abstract

Staphylococcus aureus is a major cause of morbidity and mortality worldwide. S. aureus colonizes 20 to 80% of humans at any one time and causes a variety of illnesses. Strains that are resistant to common antibiotics further complicate management. S. aureus vaccine development has been unsuccessful so far, largely due to the incomplete understanding of the mechanisms of protection against this pathogen. Here, we studied the role of different aspects of adaptive immunity induced by an S. aureus vaccine in protection against S. aureus bacteremia, dermonecrosis, skin abscess, and gastrointestinal (GI) colonization. We show that, depending on the challenge model, the contributions of vaccine-induced S. aureus-specific antibody and Th1 and Th17 responses to protection are different: antibodies play a major role in reducing mortality during S. aureus bacteremia, whereas Th1 or Th17 responses are essential for prevention of S. aureus skin abscesses and the clearance of bacteria from the GI tract. Both antibody- and T-cell-mediated mechanisms contribute to prevention of S. aureus dermonecrosis. Engagement of all three immune pathways results in the most robust protection under each pathological condition. Therefore, our results suggest that eliciting multipronged humoral and cellular responses to S. aureus antigens may be critical to achieve effective and comprehensive immune defense against this pathogen.

## Abstract (precis)

S. aureus is a leading cause of healthcare- and community-associated bacterial infections. S. aureus causes various illnesses, including bacteremia, meningitis, endocarditis, pneumonia, osteomyelitis, sepsis, and skin and soft tissue infections. S. aureus colonizes between 20 and 80% of humans; carriers are at increased risk for infection and transmission to others. The spread of multidrug-resistant strains limits antibiotic treatment options. Vaccine development against S. aureus has been unsuccessful to date, likely due to an inadequate understanding about the mechanisms of immune defense against this pathogen. The significance of our work is in illustrating the necessity of generating multipronged B-cell, Th1-, and Th17-mediated responses to S. aureus antigens in conferring enhanced and broad protection against S. aureus invasive infection, skin and soft tissue infection, and mucosal colonization. Our work thus, provides important insights for future vaccine development against this pathogen.

## INTRODUCTION

Staphylococcus aureus is a leading cause of community- and healthcare-associated bacterial infections and postsurgical wound infections (1–4). Skin and soft tissue infections (SSTIs) are a common type of community-acquired S. aureus infection, which can be recurrent in many individuals (5, 6). S. aureus also causes severe invasive disease, such as bacteremia, meningitis, endocarditis, osteomyelitis, pneumonia, sepsis, and toxic shock syndrome (4, 7). S. aureus bacteremia is associated with high mortality (20 to 40% in adults) despite appropriate antibiotic treatment (8). S. aureus colonizes about 20 to 80% of the human population at any given time, providing a reservoir for subsequent infection and transmission (9–12). The rapid increase of S. aureus strains that are resistant to multiple antibiotics, such as methicillin-resistant S. aureus (MRSA) and vancomycin-intermediate and -resistant strains (VISA and VRSA, respectively), in both community- and hospital-acquired infections (13–15), has complicated the management of these infections.

The development of S. aureus vaccines has been challenging. For diseases caused by many bacterial pathogens, such as Streptococcus pneumoniae, Haemophilus influenzae type b, and Neisseria meningitidis, antibodies to polysaccharide (PS) or protein antigens, generated by either natural exposure or immunization, are highly protective (16, 17). A similar approach has been attempted for S. aureus vaccine development but yielded disappointing results so far. While multiple candidates targeting various S. aureus PSs and/or proteins have shown promise in preclinical studies, no antibody-based S. aureus vaccine (via either passive or active immunization) has succeeded in clinical trials (18–23). This failure has then led to further deliberation about the immunological requirements for effective S. aureus defense. Indeed, despite the suggestion that individuals with high-titer preexisting anti-S. aureus antibody may have better prognosis during S. aureus bacteremia and sepsis (24–26) (while S. aureus-specific T-cell immunity in those individuals and its contribution to protection were not examined in the same studies), no direct correlation has ever been established between the level of anti-S. aureus antibody and the prevention of S. aureus infection or colonization (18, 27–29), suggesting that whatever protective role antibodies may play is insufficient to effectively prevent S. aureus pathogenesis. Furthermore, a growing body of literature now implicates the importance of cellular immunity in innate and possibly acquired S. aureus resistance. Indeed, compared to the general population, HIV-infected individuals have significantly higher rates of S. aureus SSTI, bacteremia, endocarditis, and colonization (30–33). A recent study suggests that the decreased S. aureus-specific Th1 immunity may be part of the reason for the increased incidence of MRSA SSTI in HIV patients (34). Another classic immunodeficiency associated with frequently recurrent S. aureus skin and lung infection is Job’s syndrome (i.e., hyper-immunoglobulin E syndrome), which features in defective interleukin-17 (IL-17) production (and thus, Th17 immunity) due to mutations in the *stat3* gene (35, 36). In addition to these observations in humans, studies in mice have also pointed to the importance of innate and memory T cells in resistance to S. aureus. Lin et al. showed that deficiency in gamma interferon (IFN-γ) production enhanced mouse susceptibility to S. aureus bloodstream infection (37). Brown and coworkers reported that adoptive transfer of S. aureus-specific, memory Th1 cells protected naive mice against S. aureus peritoneal infection (38). A study in severe combined immunodeficiency (SCID) mice showed that, following immunization with the S. aureus antigen IsdB, Th17 cells were critical for protection against lethal S. aureus sepsis challenge (39). Furthermore, previous exposure to S. aureus protects mice against recurrent dermonecrosis in an antibody- and Th17-dependent fashion (40). The production of IL-17 and IL-22 by innate immune cells was also found to be critically important in the control of S. aureus nasal carriage (41–43). These observations therefore suggest that host defense against S. aureus may require the involvement of several immune factors rather than humoral responses alone.

In this work, we investigate the respective roles of vaccine-induced S. aureus-specific humoral and cellular immunity in acquired protection against S. aureus under various pathological conditions, including invasive infection, SSTI (dermonecrosis and abscess), and mucosal colonization in mice. Our results show that depending on the type of S. aureus challenge, protection is mediated by different immune pathways, including antibody, Th1 or Th17 response, a combination of the two, or all of the above. Thus, compared to an antibody-based strategy, an approach that elicits all three types of immune responses to S. aureus antigens confers more robust and broad protection in mice against both S. aureus infection and carriage.

## RESULTS

### Generation of *S. aureus*-specific immune responses.

**FIG 1:**
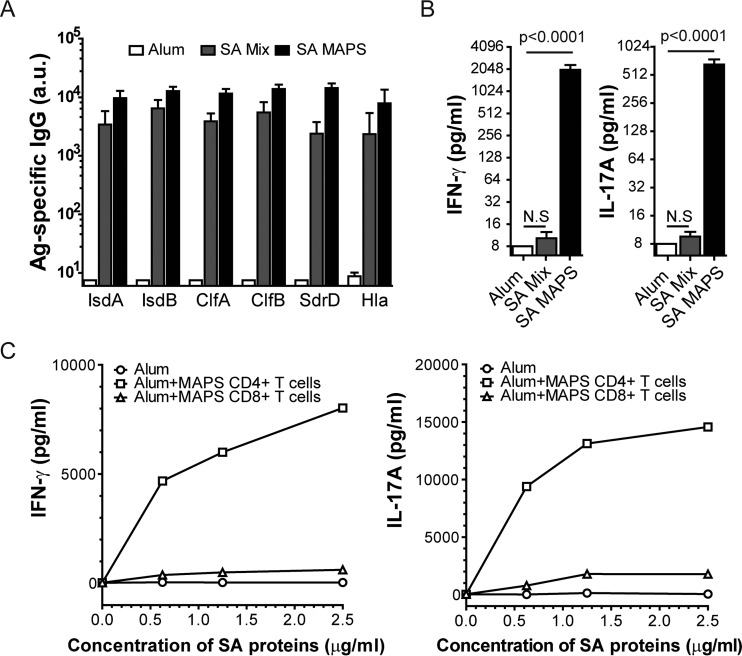
Generation of S. aureus-specific immune responses. C57BL/6 mice (*n* = 10 per group) received three subcutaneous immunizations with adjuvant alone (Alum), or Alum-adjuvanted S. aureus protein (SA) mix or S. aureus MAPS (SA MAPS) vaccine (30 µg of total protein content per dose). (A) Antigen (Ag)-specific IgG antibodies were measured 2 weeks after the third immunization. Antibody titers are expressed in arbitrary units (a.u.) related to a reference serum (as described in Materials and Methods). Bars represent geometric means + 95% confidence interval (CI). (B) IFN-γ and IL-17A production after *ex vivo* stimulation of peripheral blood cells of immunized mice with a mixture of purified S. aureus proteins. Bars represent geometric means + 95% CI. Statistical analysis was performed using nonparametric one-way ANOVA (Dunn’s multiple comparison test) between indicated groups. N.S, not significant. (C) IFN-γ and IL-17A production after *ex vivo* stimulation of splenocytes isolated from an Alum-immunized mouse with S. aureus proteins in the absence or presence of CD4^+^ or CD8^+^ T cells purified from the spleen of an S. aureus MAPS-immunized mouse.

To generate different adaptive immune responses to S. aureus antigens, we immunized C57BL/6 mice with two antigen formulations. The first formulation consisted of a mixture of six S. aureus proteins (referred to as S. aureus mix below), including α-hemolysin (Hla) toxoid (see Fig. S1 in the supplemental material), clumping factors A (ClfA) and B (ClfB), serine-aspartate repeat protein D (SdrD), and iron-regulated surface proteins A (IsdA) and B (IsdB) (see Fig. S2 in the supplemental material). When administered with aluminum hydroxide adjuvant (Alum), S. aureus mix induces robust antibodies, but no measurable cellular responses to the included antigens (Fig. 1A and B, SA mix). The second formulation consisted of a macromolecular complex (called the multiple antigen presenting system [MAPS] complex) in which the same six S. aureus proteins were coupled to a biotinylated polysaccharide scaffold via affinity interaction between rhizavidin (rhavi) and biotin molecules (44) (see Fig. S2 in the supplemental material). As shown previously with other antigens (44), immunization of mice with S. aureus MAPS complexes and Alum induced not only a high level of antibodies, but also antigen-specific adaptive cellular responses, as indicated by robust production of IFN-γ and IL-17A upon *ex vivo* stimulation of peripheral blood with the target protein antigens (Fig. 1A and B, SA MAPS). Further analysis indicated that both cytokines are primarily produced by CD4^+^ T helper cells (Fig. 1C), representing Th1 and Th17 responses.

10.1128/mBio.01949-18.1 FIG S1 Hemolytic activity of wild-type (WT) Hla, the Hla209 mutant, and their rhavi fusion proteins. One hemolytic unit (HU) was defined as the activity that causes 50% hemolysis of 1% rabbit red blood cells in PBS (pH7.5) after 30 min of incubation at 37°C. Hemolytic activity was expressed as HU per 1 mg/ml of each construct. Symbols represent means ± SEM. The dashed line indicates 50% hemolysis of red blood cells. Download FIG S1, TIF file, 0.7 MB. Copyright © 2018 Zhang et al. 2018 Zhang et al. This content is distributed under the terms of the Creative Commons Attribution 4.0 International license.

10.1128/mBio.01949-18.2 FIG S2 Preparation of S. aureus protein mixture and S. aureus MAPS complex. (A) Schematics of S. aureus protein mixture (SA Mix) and S. aureus (SA) MAPS complex. (B) Reduced SDS-PAGE of purified S. aureus MAPS complexes. S. aureus MAPS complexes retain their integrity after treatment with the reducing-SDS sample buffer at room temperature (RT) and remain at the top of SDS-PAGE gel due to their large molecular weight. The rhavi-S. aureus fusion antigens are released from the PS scaffold after S. aureus MAPS complexes were fully dissociated by boiling in SDS sample buffer (Boil). Download FIG S2, TIF file, 2.1 MB. Copyright © 2018 Zhang et al. 2018 Zhang et al. This content is distributed under the terms of the Creative Commons Attribution 4.0 International license.

### Protection against *S. aureus* is mediated by different adaptive immune responses.

**FIG 2:**
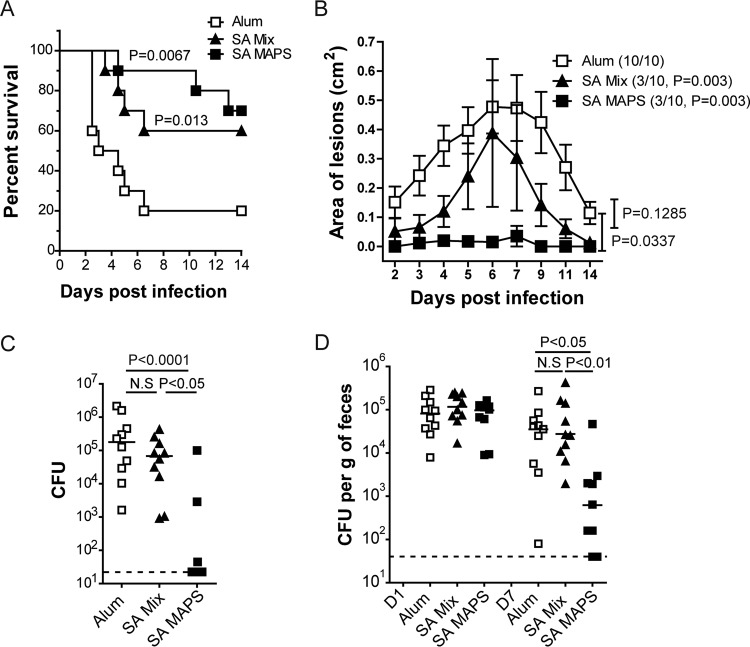
Acquired protection against S. aureus invasive disease, SSTI, and GI colonization. Mice (*n =* 10 per group) were immunized as described previously. Three weeks after the last immunization, mice were challenged in models of bacteremia (A), dermonecrosis (B), skin abscess (C), and GI colonization (D). (A) Survival curves after infection. Statistical analysis was performed by the Mantel-Cox test in comparison to the Alum group. (B) The incidence of dermonecrosis in each group is indicated in the symbol key. Individual curves show progression of lesions over time in those animals that developed dermonecrosis postinoculation. Symbols represent means ± standard errors of the means (SEM). Statistical analysis was performed using Fisher’s exact test (for incidence) or two-way ANOVA (for lesion size) in comparison to the Alum group. (C) Bacterial CFU recovered from skin abscesses 4 days postinoculation. Each symbol represents one mouse, and lines indicate medians. The dashed line indicates one-half of the lower detection limit (22.5 CFU). Statistical analysis was performed using nonparametric one-way ANOVA (Dunn’s multiple comparison test) between indicated groups. (D) Bacterial CFU recovered from feces 1 day (D1) and 7 days (D7) postinoculation. Each symbol represents one mouse, and lines indicate medians. The dashed line indicates one-half of the lower detection limit (40 CFU). Statistical analysis was performed using nonparametric one-way ANOVA (Dunn’s multiple comparison test) between indicated groups.

Acquired protection mediated by anti-S. aureus immune responses was evaluated in four challenge models. For invasive infection, we used a bacteremia model, in which mice were injected intravenously with 2 × 10^7^ CFU of S. aureus (ATCC 29213 strain, type 5 capsule-expressing). Protection was evaluated by comparing survival curves over 14 days (Fig. 2A). For SSTI, we used two different models. In the dermonecrosis model, subcutaneous inoculation of mice with 1 × 10^7^ CFU of S. aureus (USA300 TCH959 strain) leads to severe skin damage and the formation of necrotic lesions (45). Protection was assessed with respect to the overall incidence of lesions (Fig. 2B, symbol key) and the surface area of the dermonecrotic lesion in those animals that were affected (Fig. 2B, curves). In the skin abscess model, mice were infected with a lower inoculum (∼2 × 10^5^ to 5  × 10^5^ CFU), which induces enclosed subdermal abscesses with minimal skin breakdown (45). Protection was assessed by comparing densities of S. aureus recovered from abscesses dissected 4 days postinfection (Fig. 2C). Finally, S. aureus gastrointestinal (GI) colonization was examined following intranasal inoculation of mice with 5 × 10^7^ CFU of the USA300 LAC^Strep^ strain; this results in stable GI colonization in naive mice for >21 days (see Fig. S3 in the supplemental material). Protection was assessed by comparing bacterial densities in feces at indicated time points postinoculation (Fig. 2D).

10.1128/mBio.01949-18.3 FIG S3 GI colonization with S. aureus in naïve C57BL/6 mice. Naïve C57BL/6 mice (*n *=* *10) were inoculated intranasally with 5 × 10^7^ CFU of the USA300 LAC^strep^ strain. Feces were collected on days 1, 7, 14, and 21 postinoculation for CFU analysis. Each symbol represents one mouse, and lines indicate medians. The dashed line indicates one-half of the lower detection limit (40 CFU). Download FIG S3, TIF file, 0.6 MB. Copyright © 2018 Zhang et al. 2018 Zhang et al. This content is distributed under the terms of the Creative Commons Attribution 4.0 International license.

We found that mice that developed only anti-S. aureus antibodies (i.e., those in the S. aureus mix group) were significantly protected in two of the four models, with reduced mortality following intravenous (i.v.) infection (40 versus 80% in the control group [Fig. 2A]) and a decreased incidence of lesions in the dermonecrosis model (30% versus 100% in the control group [Fig. 2B]). However, with respect to skin abscess or GI colonization, they were equally susceptible as the control group (Fig. 2C and D). In contrast, mice that developed both antibody and cellular responses to S. aureus antigens (i.e., those in the S. aureus MAPS group) demonstrated broad resistance to all four S. aureus challenges, including bacteremia (reduced mortality and delayed disease onset) and dermonecrosis (reduced incidence and symptoms of lesions) (Fig. 2A and B), as well as in the abscess and GI colonization models. In the skin abscess model, 7 out of 10 S. aureus MAPS-vaccinated mice had no detectable abscess (and no recoverable bacteria) 4 days postinfection, whereas mice in the control group or the S. aureus mix group all had skin abscesses and recoverable CFU ranging from 10^3^ to 10^6^ (Fig. 2C). In the GI colonization model, with an initial inoculation density of 10^5^ CFU per g of feces (median, 1 day postchallenge), S. aureus MAPS-vaccinated mice were able to rapidly clear bacteria from the GI tract: S. aureus could not be detected in 2 out 10 mice 7 days postchallenge, and the group had a median bacterial density of 641 CFU per g of feces, 40- to 50-fold lower than bacterial densities at the same time point in the control group or the S. aureus mix group (Fig. 2D).

### Differential roles of antigen-specific antibodies and Th1 and Th17 responses in protection against *S. aureus*.

The results above suggest that adaptive humoral or cellular responses may contribute differentially to protection against S. aureus infections or colonization. Next, we dissected the role of each immune pathway in individual challenge models.

**FIG 3:**
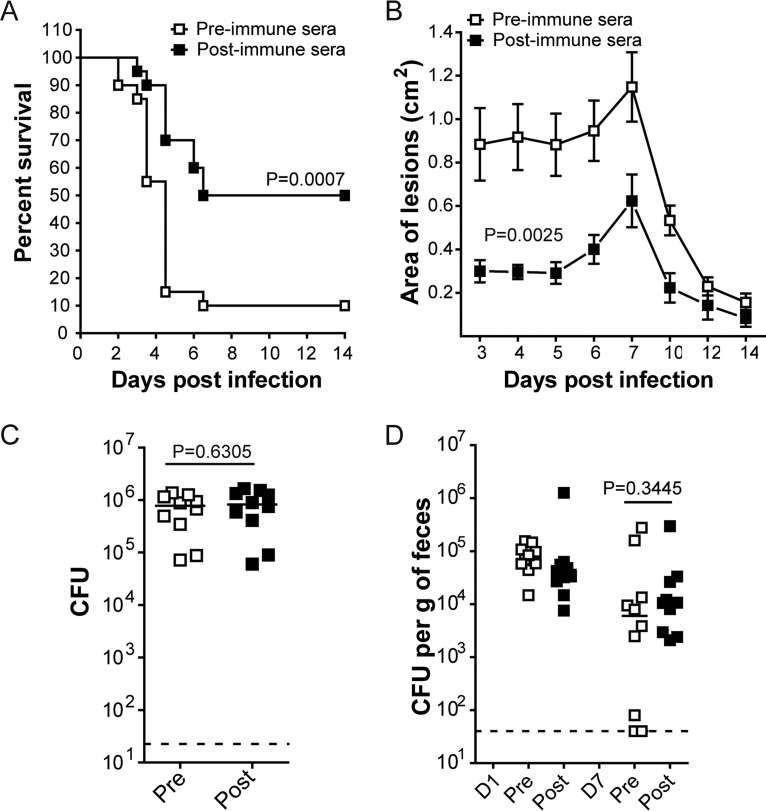
Evaluation of antibody-mediated protection against S. aureus in different models. Mice (*n* = 10 per group) received 200 µl of pre- or postimmune rabbit sera 1 day prior to S. aureus inoculation. (A) Survival curves during S. aureus bacteremia. Differences between groups were analyzed by the Mantel-Cox test. (B) Size of lesions at different time points postinoculation in the dermonecrosis model. Symbols represent mean ± SEM. Differences between the Alum and S. aureus protein (SA) mix groups or Alum and S. aureus (SA) MAPS groups were compared using two-way ANOVA. (C) Bacterial CFU recovered post-S. aureus inoculation in the abscess model. Each symbol represents one animal, and lines indicate medians. A dashed line indicates one-half of the lower detection limit (22.5 CFU). Statistical analysis was performed using the Mann-Whitney *U* test (two tailed). (D) Bacterial CFU recovered from feces 1 day (D1) and 7 days (D7) postinoculation. Each symbol represents one mouse, and lines indicate the median. A dashed line indicates one-half of the lower detection limit (40 CFU). Statistical analysis was performed using the Mann-Whitney *U* test (two tailed).

The contribution of antibodies was evaluated by passive immunization. We obtained sera from rabbits pre- or post-S. aureus MAPS vaccination (see Fig. S4 in the supplemental material) and passively transferred these to mice before challenge in each model. In the bacteremia model, mice that received postimmune sera had significantly lower mortality at 14 days postinfection compared to the control group (50% versus 90%, *P  = *0.0007) (Fig. 3A). In a separate experiment, we sacrificed the mice 20 h postinfection and measured bacterial burden in their kidneys: as shown in Fig. S5 in the supplemental material, the group that received postimmune sera had significantly lower CFU than the control group, suggesting that antibody-mediated bacterial clearance contributes to protection in this model. Passive immunization also effectively mitigated (but did not fully prevent) S. aureus dermonecrosis, resulting in reduced lesion size (Fig. 3B). However, the presence of S. aureus-specific antibodies did not provide any protection against either skin abscess or GI colonization (Fig. 3C and D).

10.1128/mBio.01949-18.4 FIG S4 Generation of rabbit sera against S. aureus protein antigens. New Zealand White rabbits received three intramuscular immunizations, 2 weeks apart, with S. aureus (SA) MAPS complex. Pre- and postimmune serum IgG antibodies directed against each S. aureus protein antigen were analyzed by ELISA. Download FIG S4, TIF file, 0.6 MB. Copyright © 2018 Zhang et al. 2018 Zhang et al. This content is distributed under the terms of the Creative Commons Attribution 4.0 International license.

10.1128/mBio.01949-18.5 FIG S5 Passive immunization of mice with S. aureus (SA) MAPS-immunized rabbit sera reduces bacterial burden in kidneys after S. aureus i.v. infection. Mice (*n *=* *15 per group) received either pre- or post-S. aureus MAPS immune rabbit sera (200 µl per mouse) 1 day prior to i.v. infection with 2 × 10^7^ CFU of the ATCC 29213 strain. Mice were sacrificed 20 h after infection, and both kidneys were dissected, homogenized, and then plated for CFU measurement. Statistical analysis was performed using the Mann-Whitney *U* test (two tailed). Download FIG S5, TIF file, 0.6 MB. Copyright © 2018 Zhang et al. 2018 Zhang et al. This content is distributed under the terms of the Creative Commons Attribution 4.0 International license.

**FIG 4:**
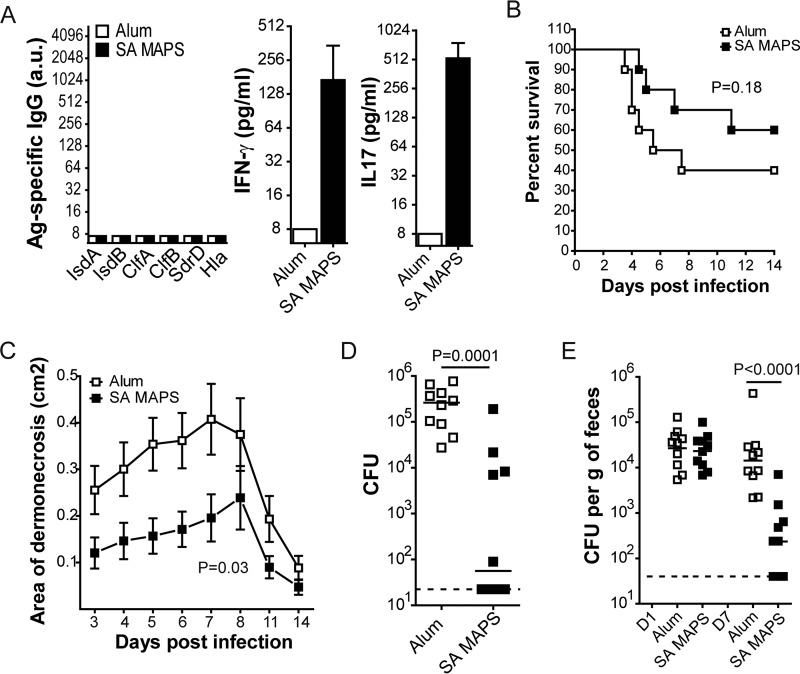
Evaluation of S. aureus MAPS-induced protection against S. aureus infection and colonization in antibody-deficient mice. µMT^−/−^ mice (*n* = 10 per group) were immunized three times with adjuvant alone (Alum) or S. aureus (SA) MAPS. (A) Antigen-specific antibody and cellular responses were examined 2 weeks after the last immunization. (B) Survival curves following intravenous infection. Differences between groups were analyzed by the Mantel-Cox test. (C) Sizes of lesions at different time points postinoculation in the dermonecrosis model. Symbols represent the mean ± SEM. Differences between groups were analyzed using two-way ANOVA. (D) Bacterial CFU recovered from skin abscesses. Lines indicate medians. Statistical analysis was performed using the Mann-Whitney *U* test (two tailed). (E) Bacterial CFU in feces 1 day and 7 days postinoculation. Lines indicate medians. Statistical analysis was performed using the Mann-Whitney *U* test (two tailed).

The contribution of cellular responses was studied in antibody-deficient (µMT^−/−^) mice. Vaccination of µMT^−/−^ mice with S. aureus MAPS induced Th1 and Th17 responses to S. aureus antigens without detectable humoral responses (Fig. 4A) and conferred significant protection in three models: compared to the control group, S. aureus MAPS-vaccinated µMT^−/−^ mice had smaller lesions during dermonecrosis challenge (*P  = *0.03 [Fig. 4C]), significantly reduced abscess formation (*P  = *0.0001 [Fig. 4D]), and accelerated clearance of S. aureus carriage from the GI tracts postcolonization (*P  < *0.0001 [Fig. 4E]). In the case of S. aureus bacteremia, the presence of only cellular responses did not provide significant protection (Fig. 4B).

Therefore, we conclude from these experiments that humoral and cellular responses serve as complementary mechanisms in providing protection against different S. aureus challenges: antibody-mediated mechanisms confer protection against S. aureus bacteremia, but are ineffective in prevention of S. aureus skin abscess or colonization, against which antigen-specific cellular responses are both essential and sufficient. Furthermore, humoral and cellular responses, collectively, confer optimal protection against S. aureus dermonecrosis.

**FIG 5:**
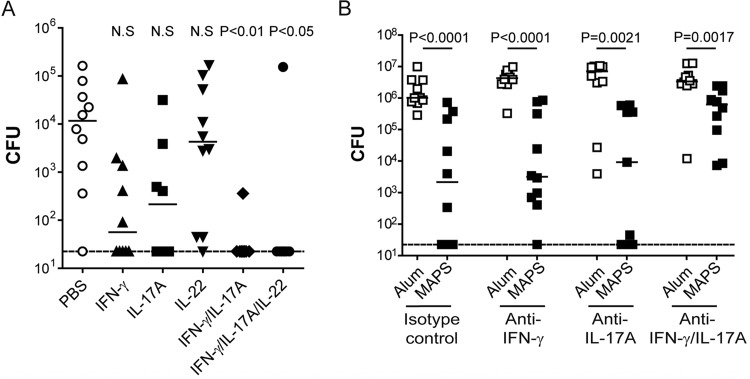
Role of Th1 and Th17 responses in prevention of S. aureus skin abscess. (A) Supplementation with recombinant mouse IFN-γ or IL-17A (but not IL-22) mitigates S. aureus abscess formation in naive mice. Mice (*n* = 8 to 10 per group) were injected subcutaneously with 2.5 × 10^5^ CFU of USA300 strain mixed with PBS (vehicle control), rIFN-γ (1 µg per mouse), rIL-17A (0.9 µg per mouse), rIL-22 (1 µg per mouse), a combination of rIFN-γ and IL-17A, or all three cytokines. Skin abscesses were dissected 4 days postinoculation for bacterial CFU quantification. Lines indicate medians. Statistical analysis was performed using nonparametric one-way ANOVA (Dunn’s multiple comparison test) in comparison to the PBS group. (B) Depletion of both IFN-γ and IL-17A significantly attenuated S. aureus (SA) MAPS-mediated protection against abscess formation. Mice (*n* = 10) were immunized three times with Alum or S. aureus MAPS before skin abscess challenge. Antibodies against IFN-γ and/or IL-17A or the isotype control were administered 1 day prior to inoculation and also on the day of inoculation. Lines indicate medians. Statistical analysis was performed between the indicated groups using the Mann-Whitney *U* test (two tailed).

As immunization with S. aureus MAPS construct induces at least two types of cellular responses, Th1 and Th17 responses, we sought to further dissect their respective contributions to protection in the abscess or GI colonization model, using cytokine supplementation or depletion approaches. To evaluate the role of Th1 or Th17 immunity against skin abscesses, we infected naive mice with S. aureus inocula that were premixed with recombinant mouse IFN-γ, IL-17A, IL-22, or a combination of different cytokines. The presence of recombinant cytokine(s) did not affect the viability of S. aureus in the inoculum (see Fig. S6 in the supplemental material). Mice in the control group were infected with S. aureus mixed with buffer vehicle: 9 out of 10 animals (90%) developed skin abscesses with a median bacterial density of >10^4^ CFU (Fig. 5A, phosphate-buffered saline [PBS]). In contrast, supplementation of the inoculum with either IFN-γ or IL-17A but not IL-22 during infection was able to lower the incidence of abscess to 50% and reduce the median bacterial burden to 55 or 208 CFU, whereas coadministration of both IFN- γ and IL-17A resulted in almost complete protection (9/10 mice were free of abscess 4 days postinfection) (Fig. 5A). This result was further confirmed by cytokine depletion in S. aureus MAPS-vaccinated animals during abscess challenge, which showed that S. aureus MAPS-induced protection was only slightly impacted by depletion of either IFN-γ or IL-17, but was significantly attenuated when antibodies to both cytokines were administered (Fig. 5B). We noticed that protection was not completely abolished by administration of anti-IFN-γ and anti-IL-17 antibodies. This result may be due to an insufficient concentration of administered antibodies and/or the inability of antibodies to effectively access and neutralize locally produced cytokines at the site of abscess formation. Taken together, these results suggest that either Th1 or Th17 responses to these antigens may be sufficient to prevent S. aureus skin abscess.

10.1128/mBio.01949-18.6 FIG S6 The presence of recombinant mouse cytokines does not affect S. aureus growth *in vitro*. A total of 2.5 × 10^5^ CFU of the USA300 strain (in 100 µl) were mixed with PBS, rIFN-γ (1 µg), rIL-17A (0.9 µg), rIL-22 (1 µg), a combination of rIFN-γ and IL-17A, or all three cytokines and incubated at room temperature for 2 h before dilution with saline and plating on mannitol salt plates. (Each condition was performed in duplicate.) Bacterial CFU were measured after overnight incubation of plates at 37°C. Download FIG S6, TIF file, 0.6 MB. Copyright © 2018 Zhang et al. 2018 Zhang et al. This content is distributed under the terms of the Creative Commons Attribution 4.0 International license.

**FIG 6:**
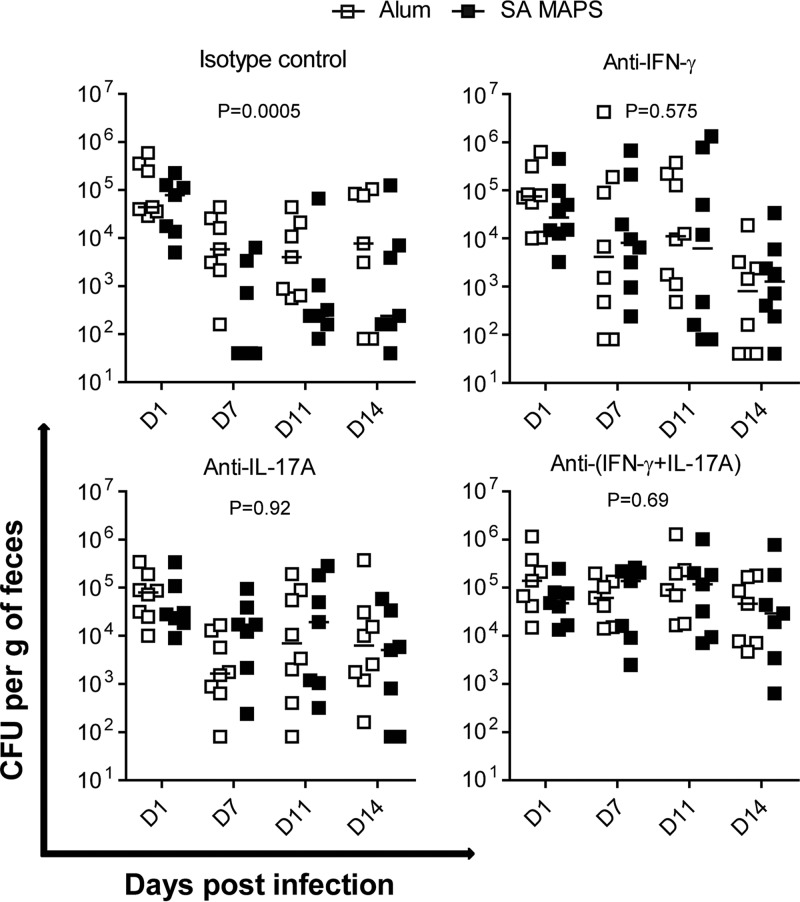
Role of Th1 and Th17 responses in clearance of S. aureus GI colonization. Mice (*n* = 10) were immunized three times with Alum or S. aureus (SA) MAPS prior to challenge in the GI colonization model. Antibodies against IFN-γ and/or IL-17A or the isotype control were administered 1 day prior to inoculation and also on days 1, 5, and 8 postinoculation. Feces were collected on days 1, 7, 11, and 14 postinoculation for CFU analysis. Lines represent medians. Statistical analysis was performed using two-way ANOVA.

In contrast to what was observed in the abscess model, when a cytokine depletion was performed in the GI colonization model we found that depletion of either IFN-γ or IL-17 completely abolished S. aureus MAPS-induced protection in mice, suggesting that the clearance of S. aureus carriage may require both Th1- and Th17-mediated immune responses (Fig. 6).

## DISCUSSION

Humoral immunity and cellular immunity are two important arms of host defense against microbial invasions. While the strategy of generating antibodies has been widely used in bacterial vaccinology and highly successful in the prevention of several diseases, there is a growing recognition that adaptive cellular responses, such as Th1 and Th17 responses, may play a critical role in protection against infection and/or colonization by certain extracellular bacterial pathogens (46). Studies in mice showed that Th17 memory responses specific to S. pneumoniae could facilitate nasopharyngeal clearance (47, 48) and provide cross-serotype protection against invasive pneumococcal disease (49). Moreover, a recent work using a nonhuman primate model revealed an important role of Th17/Th1 memory generated by the whole-cell pertussis vaccine in protection against colonization, transmission, and secondary infection of Bordetella pertussis and hypothesized that the absence (or significant reduction) of such cellular responses induced by current acellular pertussis vaccines may be an important reason for their inferior efficacy (50).

The development of S. aureus vaccines has been extremely challenging. In addition to the wide variety of diseases for which the organism is responsible, S. aureus is a common colonizer of healthy individuals, with a concomitant risk of transmission and skin and soft tissue autoinoculation. It is unclear whether natural immunity to S. aureus truly occurs: unlike for other common bacterial pathogens such as pneumococcus, Haemophilus influenzae type b, and meningococcus, there is no clear age association with S. aureus infections, and recurrence is not uncommon. These findings suggest that the naturally induced immune responses to S. aureus infections, antibodies (51–54) and T-cell responses (38, 55), are likely insufficient to protect against this pathogen. Previous preclinical studies of S. aureus vaccine candidates had primarily focused on invasive infection models in which antibodies to specific S. aureus polysaccharide and/or protein antigens were found to be highly protective. However, the failure of all antibody-based vaccine candidates in clinical trials to date could be interpreted as indicating that immune mechanisms other than antibodies may be required to effectively reduce or prevent S. aureus pathogenesis in humans. Another important implication is that preclinical evaluation using different challenge models, including invasive infection, SSTI, and mucosal colonization models, in which protection is mediated via various immune factors (e.g., antibodies and Th1 and/or Th17 responses) (38, 40, 43), may be important to properly assess the potential of vaccine candidates.

In this work, we elucidate the importance of vaccine-induced S. aureus-specific antibody and Th1 and Th17 responses in conferring comprehensive protection against S. aureus infection and colonization. Our results show that different immune responses mediate protection depending on the site and type of S. aureus infection/colonization. For instance, antibodies protect mice against S. aureus i.v. infection, and to some extent, dermonecrosis, but are ineffective in the prevention of skin abscess or GI colonization, whereas antigen-specific cellular (Th1 and Th17) responses are critically involved in protection against SSTI (dermonecrosis and abscess) and GI carriage but do not provide significant protection following i.v. infection. In this light, it is not surprising that we find optimal protection against S. aureus when all three immune pathways are engaged during vaccination. Therefore, our data strongly suggest that vaccine strategies aiming to induce multipronged B- and T-cell responses to S. aureus antigens may be critical to prevent different aspects of S. aureus pathogenesis and thus provide comprehensive protection against this pathogen.

## MATERIALS AND METHODS

### Mouse and bacterial strains.

Wild type and µMT^−/−^ C57BL/6 mice were purchased from Jackson Laboratories. S. aureus strains USA300 (TCH959) and ATCC 29213 were purchased from ATCC. The USA300 LAC (JE2) strain was kindly provided by BEI (56). The streptomycin-resistant USA300 LAC (JE2) strain (USA300 LAC^strep^) was obtained by spontaneous mutation after culturing the parent strain on a blood agar plate containing 0.5 g/liter streptomycin.

### Ethics statement.

All procedures involving mice were approved by the Boston Children’s Hospital Animal Care and Use Committee (IACUC protocol no. 16033133), following the National Institutes of Health guidelines for animal housing and care.

### Cytokines and antibodies.

Recombinant mouse IFN-γ, IL-17A, and IL-22 were purchased from R&D systems. Cytokines were reconstituted at 100 mg/ml in PBS and then diluted with PBS to the appropriate concentration during infection. Anti-mouse IFN-γ (clone R4-6A2), anti-mouse IL-17A (clone 17F3), and the corresponding isotype control antibodies were purchased from Bioxcell. For cytokine depletion in the skin abscess model, mice received 300 µg of anticytokine antibodies or isotype control via intraperitoneal injection 1 day prior to infection and another 300 µg via subcutaneous injection the same day of infection but at a distinct location from the infection site. For cytokine depletion in the GI colonization model, mice received 300 µg of the indicated antibodies via intraperitoneal injection 1 day prior to inoculation and on days 1, 5, and 8 postinoculation.

### Cloning and purification of *S. aureus* antigens.

DNA sequences encoding ClfA (positions 221 to 559), ClfB (203 to 542), SdrD (246 to 682), IsdA (47 to 324), IsdB (48 to 477), or Hla (27 to 319) were amplified from S. aureus genomic DNA (USA300 TCH959 strain) via PCR and then cloned into a pET-21b vector. A nonhemolytic toxoid of Hla was generated by substitution of residues Asp-Arg-Asp (209 to 211) to Ala-Ala-Ala using PCR. For rhizavidin fusion proteins, DNA sequences encoding the above S. aureus antigens were inserted at the 3′ end of the gene encoding the rhizavidin moiety in a pET-21b vector. All constructs were transformed into the E. coli BL21(DE3) strain for expression under isopropyl-β-d-thiogalactopyranoside (IPTG) induction. His-tagged recombinant proteins were purified using nitrilotriacetic acid (NTA) affinity chromatography (Qiagen) followed by size exclusion chromatography using a Superdex 200 column (GE Healthcare Life Sciences). Purified proteins were stored at −80°C until use.

### Preparation of *S. aureus* MAPS complexes.

Type-1 pneumococcal capsular polysaccharide was purchased from ATCC and used as the scaffold for *S. aureus* MAPS constructs. The polysaccharide was biotinylated using CDAP (1-cyano-4-dimethylaminopyridinium tetrafluoroborate) as the activation reagent as described previously (44). S. aureus MAPS complex was assembled by incubation of biotinylated polysaccharide with a mixture of rhizavidin fusions of S. aureus antigens (at equal molarity) at room temperature overnight. The input ratio of total proteins to polysaccharide was 3:1 (wt/wt). The assembled complex was isolated by size exclusion chromatography and concentrated by ultrafiltration. The protein concentration of S. aureus MAPS complexes was measured using a bicinchoninic acid (BCA) protein assay kit (Thermo Scientific). The incorporation of S. aureus antigens was examined on a reduced SDS-PAGE gel.

### Immunization and infection.

All vaccines were formulated the day prior to immunization. The antigens were diluted to the appropriate concentration in saline and then mixed with aluminum hydroxide (1.25-mg/ml final concentration [Brenntag]) in 5-ml Eppendorf tubes and incubated at 4°C overnight with rotation (24 rpm) on a Mini LabRoller (Labnet International, Inc.). Four- to 6-week-old female mice received three subcutaneous immunizations on the upper back (30 µg of total protein per immunization per mouse in a 200-µl volume) 2 weeks apart. Animals were bled under isoflurane anesthesia 2 weeks after the last immunization for measurement of antibody and analysis of T-cell responses. The endotoxin concentration in the S. aureus mix and MAPS vaccine were 23 and 11 EU per dose, respectively, as measured using the Pierce Chromogenic Endotoxin Quant kit (Thermo Scientific).

For passive immunization experiments, we used rabbit antisera, based on the established finding that rabbit IgG is compatible with mouse Fcγ receptors (57) and to obtain adequate volumes of sera. Rabbit anti-S. aureus MAPS sera were generated at Cocalico Biologicals (Reamstown, PA). New Zealand White rabbits were given three intramuscular immunizations, 2 weeks apart, with S. aureus MAPS vaccine (300 µg of total protein per immunization per rabbit in 500-µl volume). Sera were collected before the first immunization (preimmune sera) and 2 weeks after the last immunization (antisera). Antigen-specific IgG antibody was detected by enzyme-linked immunosorbent assay (ELISA), and the rabbit serum with highest antibody titer was used for passive immunization in mice. For passive immunization, 8-week-old female mice received 200 µl of heat-inactivated pre- or postimmune sera 1 day prior to infection via intraperitoneal injection.

For preparation of inocula, S. aureus strains were streaked onto blood agar plates and grown at 37°C. Colonies were picked and inoculated into tryptic soy broth (TSB [Sigma]) for an overnight culture. In the following morning, bacteria were reinoculated into fresh TSB medium at a 1:100 dilution and incubated at 37°C with shaking. Bacteria were collected 3 h later by centrifugation, washed twice with saline, and adjusted to the appropriate concentration in saline before infection.

Mice were infected 3 weeks after the last immunization. The bacteremia model was performed using the ATCC 29213 strain as described previously (58) with minor modifications. Briefly, mice were anesthetized with isoflurane and injected intravenously with 2 × 10^7^ CFU in 100 µl. Mice were monitored for any sign of illness for 14 days; any ill-appearing animal (presenting with signs of ruffled fur, slow moving, and/or with closed eyes) was immediately and humanely euthanized. In the dermonecrosis model, mice were anesthetized and injected subcutaneously on the shaved lower back with 1 × 10^7^ CFU of the USA300 strain in a 100-µl volume. Mice were monitored for 14 days after infection. Pictures of the infected area were taken at different time points, and the sizes of dermonecrotic lesions were measured using ImageJ software. In the skin abscess model, the backs of mice were shaved, anesthetized, and infected subcutaneously with 2.5 × 10^5^ CFU of the USA300 strain in a 100-µl volume. Mice were then humanely euthanized 4 days after infection. Abscesses were dissected and homogenized in 500 µl of PBS using a bead beater. Serial dilutions of homogenate were plated on mannitol salt plates, and colonies were counted after overnight incubation at 37°C. For animals that were abscess free or for culture-negative samples, the CFU was arbitrarily set as one-half of the lower detection limit (22.5 CFU) to allow for statistical analysis. In the GI colonization model (59), mice were gently restrained and inoculated intranasally with 5 × 10^7^ CFU of the USA300 LAC^strep^ strain in a 10-µl volume. Fecal pellets were collected on days 1 and 7 after inoculation or as indicated. Samples were weighed, resuspended in sterile PBS at 0.1 g/ml, homogenized, and then passed through a CellTrics 30-µm-pore filter. Serial dilutions of the flowthrough samples were plated on mannitol salt plates containing 0.5 mg/ml streptomycin, and colonies were counted after overnight culture at 37°C. For culture-negative samples, CFU was set as one-half of the lower detection limit (40 CFU).

### Antibody and T-cell response analysis.

Antigen-specific IgG antibody was measured by ELISA using Immulon 2 HB 96-microwell plates (Thermo Scientific) coated with individual recombinant S. aureus protein (not containing rhizavidin) (1 µg/ml in PBS, incubated at room temperature overnight). The plates were washed with PBS containing 0.05% Tween 20 (PBS-T) and then blocked with 1% bovine serum albumin (BSA) in PBS for 1 h. After blocking, serial dilutions of mouse or rabbit sera were added and incubated for 2 h, followed by a 1-h incubation with horseradish peroxidase (HRP)-conjugated secondary antibody against mouse or rabbit IgG. The plates were then washed and developed with SureBlue TMB Microwell peroxidase substrate (KPL). HCl (1 M) was used to terminate the reactions before the *A*_450_ was analyzed using an ELISA reader. A reference serum was generated by pooling sera from 10 mice that have been immunized three times with S. aureus MAPS vaccine. The IgG titer of the reference serum against each target S. aureus protein was assigned a value of 12,000 arbitrary units per ml. Duplicates of 7 serial dilutions of the reference serum were included in each ELISA. The antibody titer of each sample serum was expressed as calculated arbitrary units per ml using standard curves generated by dilutions of the reference serum.

Antigen-specific T memory responses were analyzed by *ex vivo* stimulation of peripheral blood cells or splenocytes with purified S. aureus protein antigens (representing a recall response). For whole-blood stimulation, 25 µl of heparinized blood from each mouse was added to 225 µl Dulbecco’s modified Eagle’s medium (DMEM [BioWhittaker]) containing 10% low-endotoxin defined fetal bovine serum (FBS [HyClone]), 50 µM 2-mercaptoethanol (Sigma), and ciprofloxacin (10 µg/ml [Cellgro]). Cultures were incubated at 37°C for 6 days in the presence of 2.5 μg/ml of the mixture of six S. aureus protein antigens (equal weight ratio, non-rhizavidin containing). Supernatants were collected following centrifugation and analyzed by ELISA for IFN-γ and IL-17A concentrations (R&D Systems). Splenocytes were isolated from mice in the Alum- or S. aureus MAPS-immunized group. Splenocytes from an S. aureus MAPS-vaccinated mouse were then divided into two equal fractions, and from each fraction, CD4^+^ or CD8^+^ T cells were purified using a CD4^+^ or CD8^+^ selection kit from Miltenyi Biotec. For stimulation, 2.25 × 10^6^ splenocytes of Alum-vaccinated mouse, alone or mixed with 6 × 10^5^ purified CD4^+^ T cells or 4 × 10^5^ CD8^+^ T cells that originated from an S. aureus MAPS-immunized mouse, were added into each well of a 96-well microplate (250 µl per well) and incubated at 37°C for 3 days in the absence or presence of S. aureus proteins at the indicated concentrations. Supernatants were then collected and analyzed for cytokine production.

### Hemolysis analysis.

The hemolytic activity of wild-type Hla and Hla209 and their respective rhizavidin fusions was measured as follows. Two hundred microliters of heparinized rabbit blood was washed with cold PBS three times. Red blood cells were then resuspended in 10 ml of cold PBS (2% rabbit red blood cells), and 100 µl of a 2-fold serial dilution of Hla samples in PBS with 0.1% BSA, starting from 100 µg/ml, was added to a V-bottom 96-well plate before the addition of 100 µl of red blood cells to each well. PBS containing 0.1% Triton X-100 was used as a positive control (100% hemolysis), and PBS with 0.1% BSA was used as a negative control (0% hemolysis). The plate was incubated at 37°C for 30 min and then subjected to centrifugation at 800 × *g* for 5 min. The supernatants were transferred into a flat-bottom 96-well plate, and the *A*_545_ was measured by an ELISA reader. One hemolytic unit (HU) was defined as the activity that causes 50% lysis of 1% rabbit red blood cells after 30 min of incubation at 37°C. The activity of each Hla construct was expressed as the HU of 1 mg/ml of purified protein.

### Statistical analysis.

All statistical analyses were done using PRISM (version 5.01 for Windows, GraphPad Software, Inc.). Survival curves were analyzed by the Mantel-Cox test. Incidence of dermonecrosis was compared by Fisher’s exact test. Development of dermonecrosis postinfection was compared by two-way analysis of variance (ANOVA). Cytokine production and CFU counts in abscesses or in feces were compared using the Mann-Whitney *U* test (two-tailed), nonparametric one-way ANOVA (Dunn’s multiple comparison test), or two-way ANOVA, as indicated.
