# Supplementary figures and images for: Protection against Staphylococcus aureus Colonization and Infection by B- and T-Cell-Mediated Mechanisms
Source: mBio. 2018 Oct 16;9(5):e01949-18. doi: 10.1128/mBio.01949-18 (PMC6191547; doi:10.1128/mBio.01949-18)

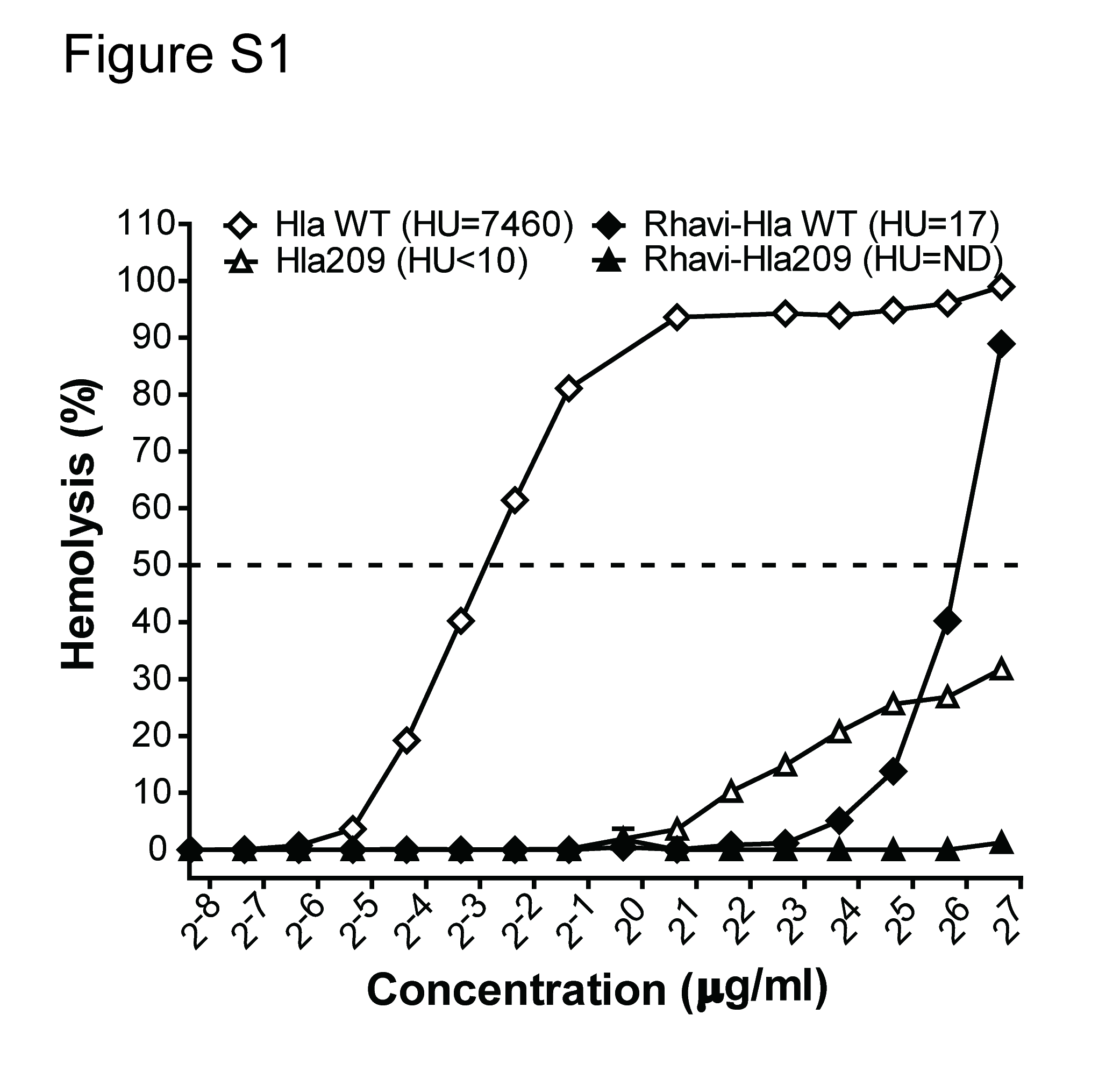

Supplement: FIG S1 [file mbo005184115sf1.tif]

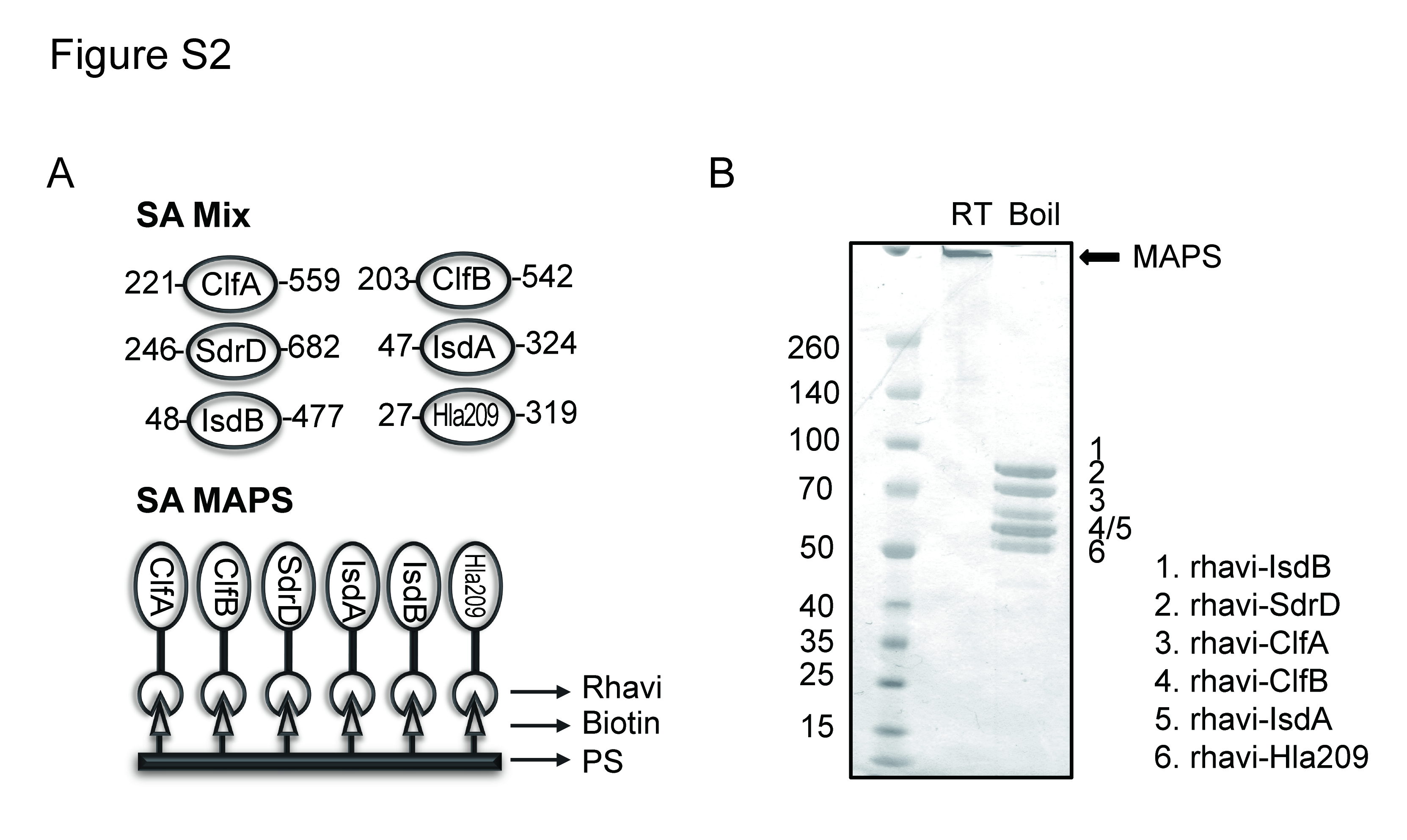

Supplement: FIG S2 [file mbo005184115sf2.tif]

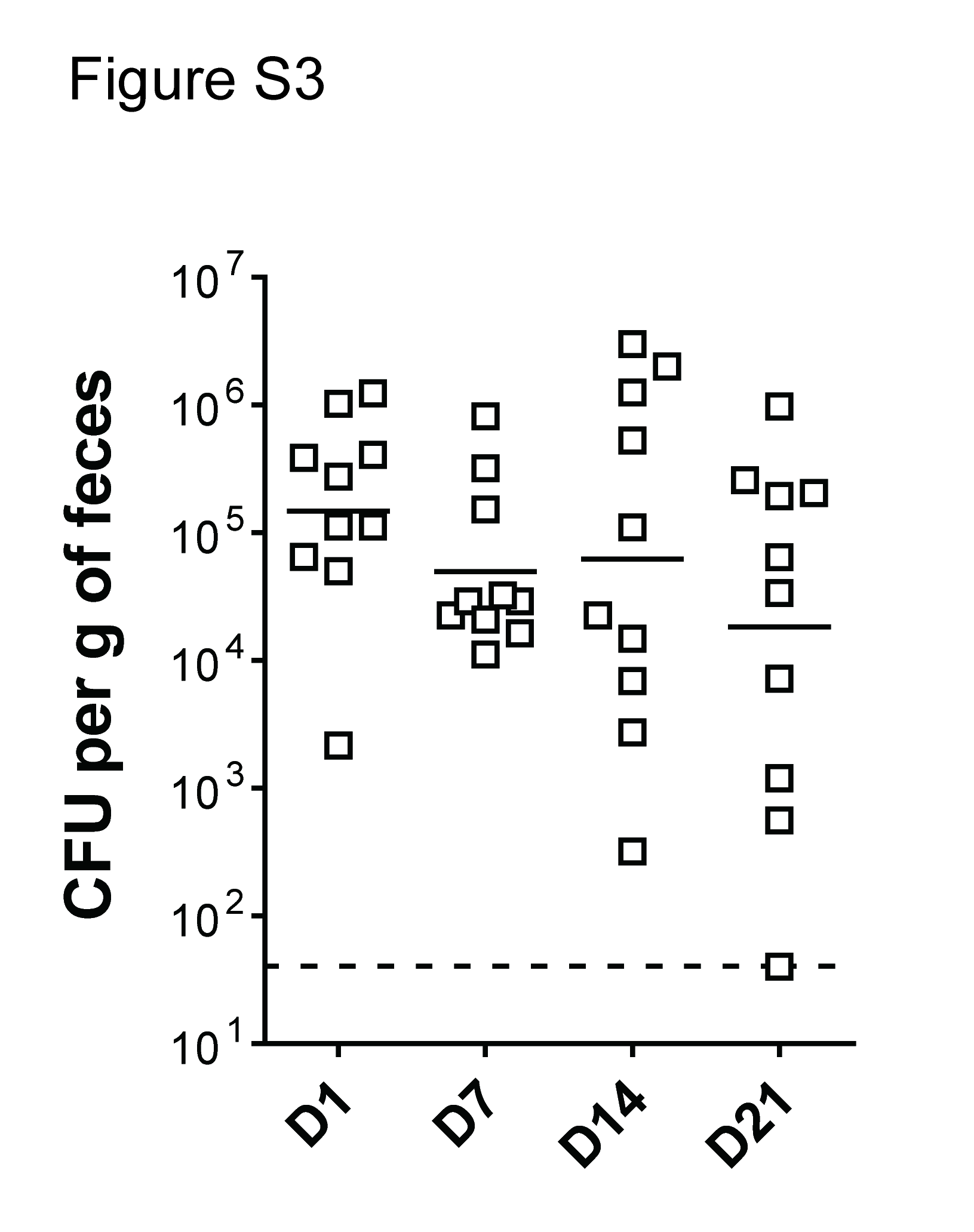

Supplement: FIG S3 [file mbo005184115sf3.tif]

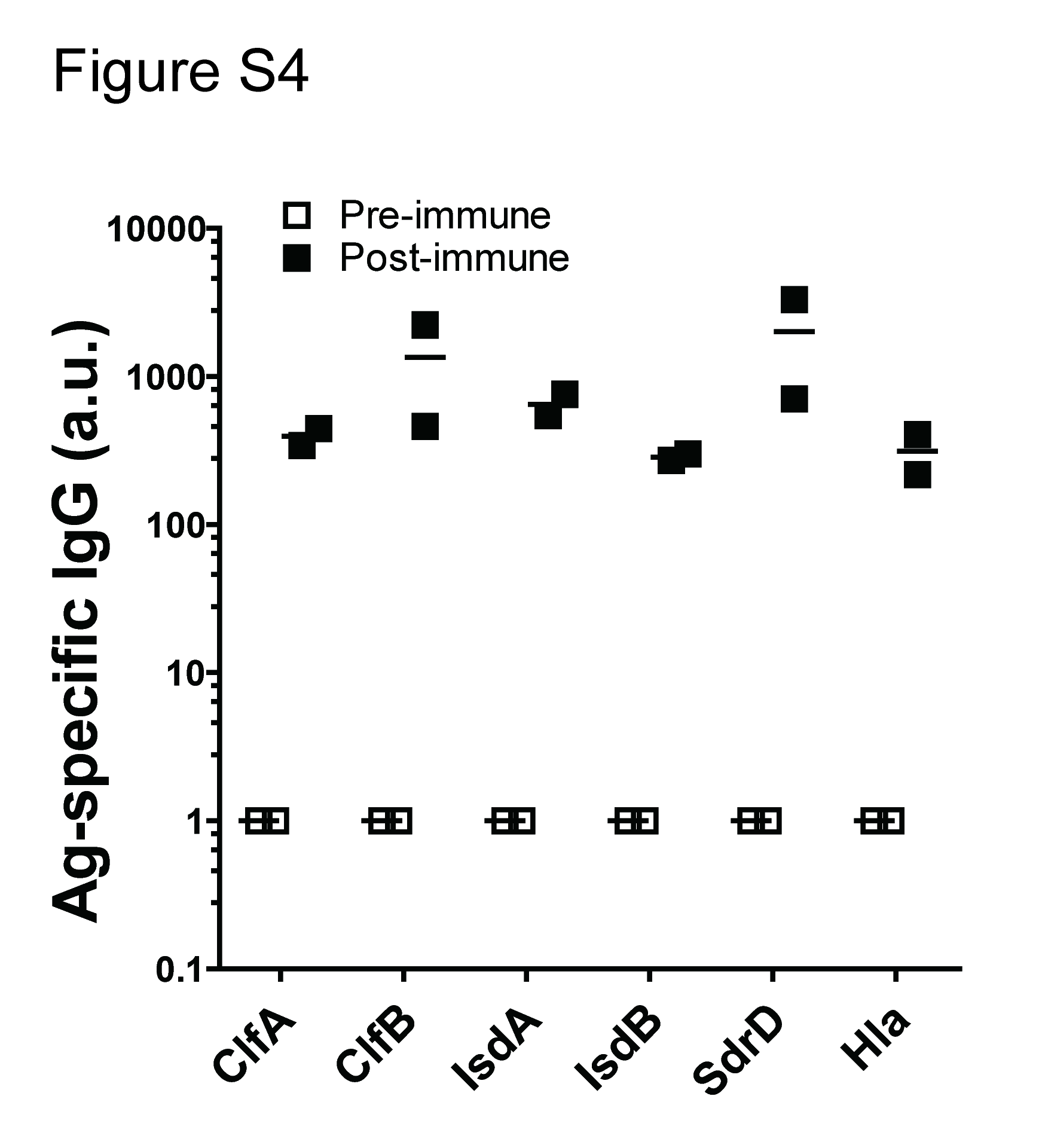

Supplement: FIG S4 [file mbo005184115sf4.tif]

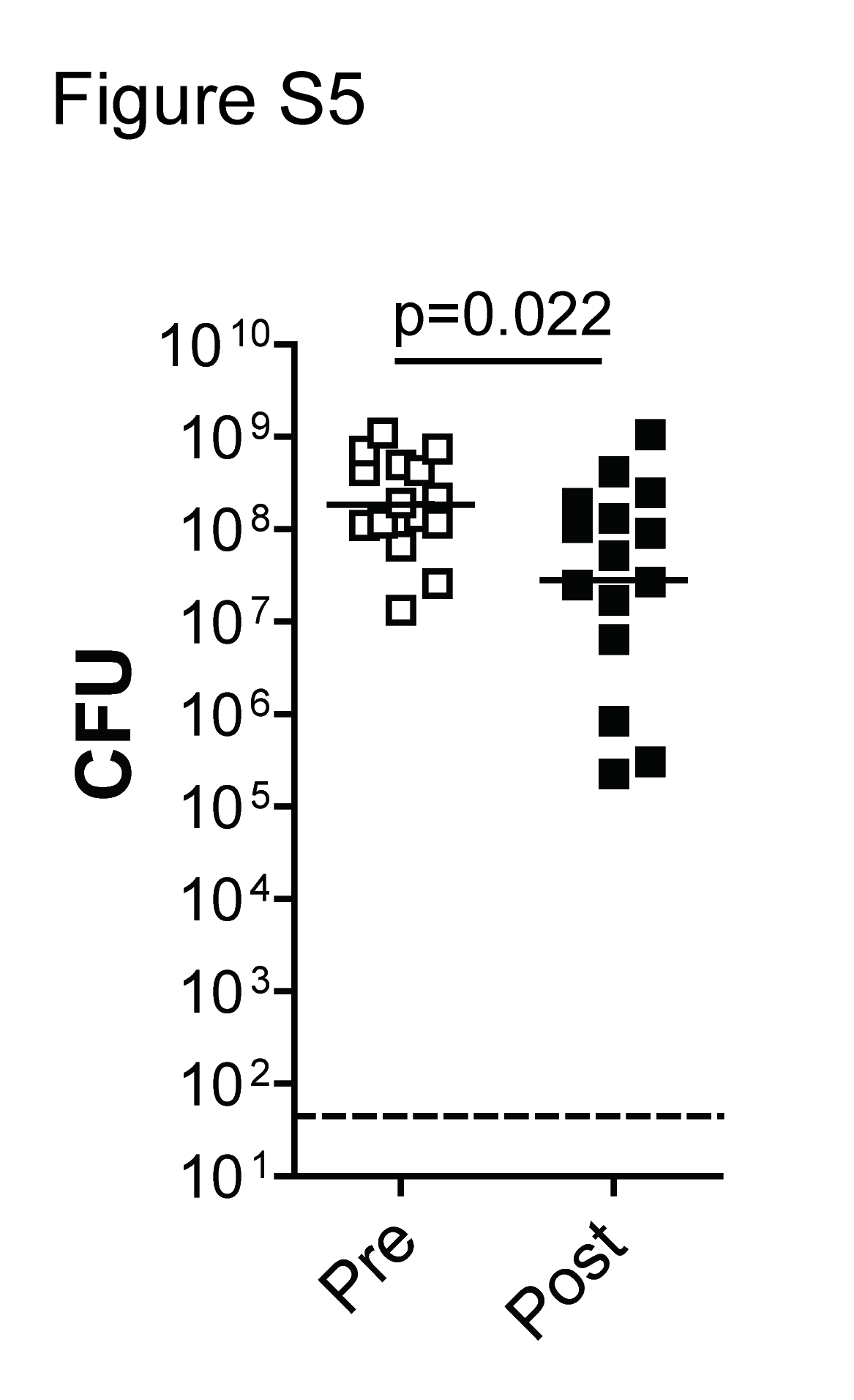

Supplement: FIG S5 [file mbo005184115sf5.tif]

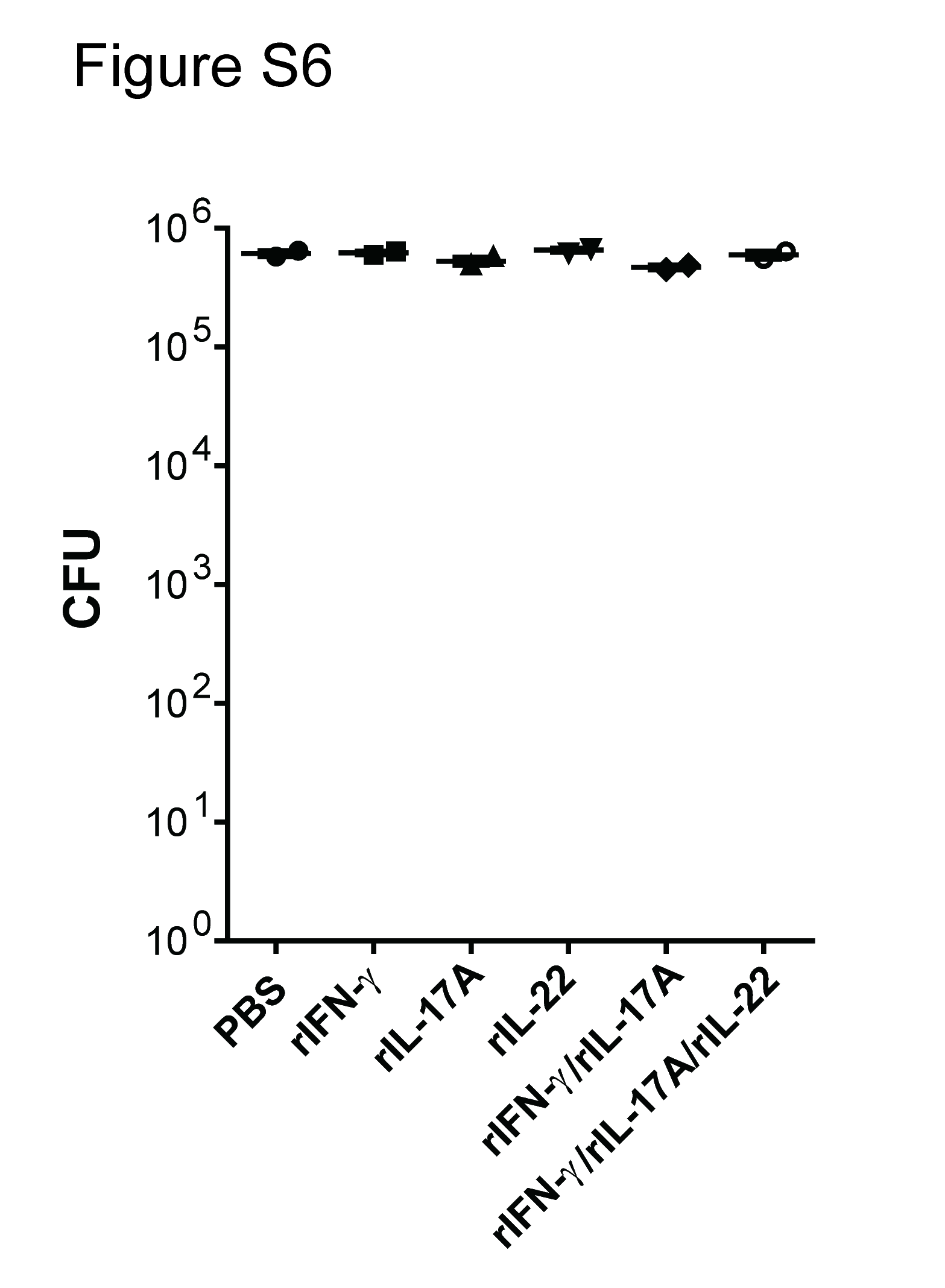

Supplement: FIG S6 [file mbo005184115sf6.tif]
